# Vector control in China, from malaria endemic to elimination and challenges ahead

**DOI:** 10.1186/s40249-022-00971-3

**Published:** 2022-05-13

**Authors:** Xinyu Feng, Jun Feng, Li Zhang, Hong Tu, Zhigui Xia

**Affiliations:** 1 National Institute of Parasitic Diseases, Chinese Center for Disease Control and Prevention (Chinese Center for Tropical Diseases Research), NHC Key Laboratory of Parasite and Vector Biology, WHO Collaborating Centre for Tropical Diseases, National Center for International Research On Tropical Diseases, Shanghai, 200025 China; 2 Shanghai Municipal Center for Disease Control and Prevention, Shanghai, 200336 China

**Keywords:** Malaria, Endemic, Vector control, *Anopheles*, Mosquito, Elimination, China

## Abstract

**Background:**

Vector control is an important approach to preventing and controlling malaria. From the malaria epidemic to malaria elimination in China, vector control has played an essential and irreplaceable role in the historical process. This review systematically summarizes the evolution, adjustment, and optimization of vector control strategy towards elimination and discusses the challenges ahead.

**Main text:**

This review first summarizes the evolution of vector control strategies during different stages of malaria epidemic, control, elimination, and post-elimination in China. We then distill the vector control experience and lessons in different stages. We discuss the current and future challenges and propose future research directions and developments for novel malaria vector control strategies.

**Results:**

Vector control has played an invaluable role in achieving malaria elimination. China adopted different prevention and control measures in response to the different malaria-endemic situations and vector distributions. Firstly, baseline surveys were initiated to establish the entomological data and helped clarify the prevention priorities and targets. Secondly, targeted and adjusted vector control strategies were conducted in various regions according to the local epidemic characteristics and different vector species. Thirdly, scientific research facilitated efficient vector-control strategies. In addition, the overall economic and social development have promoted environmental improvement, personal protection, and health care. Prediction of the vector distribution was integrated into risk assessment strategies, allowing for sustaining achievements in risk areas.

**Conclusions:**

The tailored and adapted vector control strategies have played a critical role in China’s malaria prevention, control, and elimination. Achievements and lessons learned on vector control from this progress would provide a practical reference in coping with the challenges and potential barriers other countries face in the global effort to eliminate malaria.

**Supplementary Information:**

The online version contains supplementary material available at 10.1186/s40249-022-00971-3.

## Background

Malaria remains one of the most serious public health problems and one of the most important targeted infectious diseases in the *UN Millennium Development Goals (MDGs) and Sustainable Development Goals (SDGs)* [1]. According to the latest *World Malaria Report* released by the World Health Organization (WHO) on 6 December, 2021, there were 241 million malaria cases worldwide, with an estimated death toll of 627,000 in 2020 [2]. In China, malaria has prevailed for more than 3000 years in many provinces/municipalities/autonomous regions [3]. At least thirty million malaria cases occurred, with approximately 1% of fatality rate, annually before 1949. After several generations of efforts, both the number of malaria cases and the number of areas with malaria endemics were reduced significantly. In response to the global malaria eradication initiative listed in the *United Nations Millennium Development Goals*, the Chinese government launched a national effort in 2010, with an ambitious goal to eliminate malaria in most areas by 2015 and achieve elimination across the country by 2020 [4]. In 2021, China was officially certified as malaria-free by WHO.

Malaria control requires an integrated approach, including effective prevention, early diagnosis, and prompt treatment. In addition to antimalaria drugs, vector control is a vital component in the process of malaria prevention, control, and elimination [5, 6]. Controlling the *Anopheles* vector can effectively reduce the prevalence and mortality of malaria. Traditional vector control strategies mainly include indoor residual spraying (IRS) and long-lasting insecticidal nets (LLINs) [7, 8], while biological, genetic, and environmental control strategies are also under investigation [9]. Vector control has played an essential role in each stage, from the malaria epidemic to elimination, in China [10–12]. This article summarizes the evolution of vector control strategies from the malaria epidemic stage to the control stage to the elimination and post-elimination stages in China. We also review the vector control experience and lessons learned from different stages and discuss the challenges for the future research and development of novel malaria vector control strategies.

## Main text

### The malaria vectors in China and species-specific vector control strategy

Malaria epidemics or outbreaks were more common in areas where residents had poor mosquito control and tended to sleep outside in summer and autumn, which resulted in an increased chance of contact with mosquitoes [13, 14]. There are approximately 60 species of *Anopheles* mosquito identified in China. Among them, *Anopheles sinensis*, *An. anthropophagus*, *An. minimus*, and *An. dirus* were the four major malaria vector species (Additional file 1: Fig. S1). There were also some other potential vectors, such as *An. jeyporiensis cndoidiensis*, *An. Messeae, An. pseudowillmori* and *An. willmori*, etc.

*An. sinensis* was the dominant species in most areas and was the most widely distributed malaria vector in China [12]. *An. sinensis* was the most prominent malaria vector in the plain regions, especially rice-planting areas. *An. sinensis* sucks both human and animal blood and tends to be zoophilic. The species was known to be an effective vector for *Plasmodium vivax* malaria because it was the only primary vector in the endemic regions of central China [15]. However, *An. sinensis* was not an highly efficient vector for malaria transmission, since its sporozoite infection rate was only between 0.01% and 0.93% [12, 16]. Since the feeding preferences of *An. sinensis* were mainly zoophilic [usually human blood index (HBI) < 0.3] and its larvae mainly breed in rice fields, the corresponding vector control is carried out by indoor residual spraying of animal enclosures combined with intermittent irrigation and biological control measures.

*An. anthropophagus* was distributed between 25°N and 33°N, with a distribution range of 245 counties in 18 provinces, as well as in northern regions such as Liaoning. *An. anthropophagus* was an important vector for malaria transmission [17, 18]. Historical data have shown that *An. anthropophagus* was the main vector for malaria outbreaks, with a vector capacity more than ten times that of *An. sinensis* in the same region. The natural sporozoite infection rate was generally above 0.4%, while in *falciparum* malaria experiments, the sporozoite infection rate was approximately 10.9% [19], 20]. In addition, multiple surveys have shown that a higher malaria prevalence was commonly found in areas where *An. anthropophagus* and *An. sinensis* coexisted than in areas where only *An. sinensis* was identified. The results of malaria vector surveillance in recent years showed that the distribution range of *An. anthropophagus* was significantly reduced [21, 22]. At present, only a few areas in Yunnan, Guizhou, and Liaoning have reported the distribution of *An. anthropophagus*. Given that this species was of high vector capacity, insecticide treated nets (ITNs) or LLINs and IRS were two combined important tools for vector control.

*An. minimus* was distributed in the hilly areas of China (mainly below 33°N latitude, and even 25°N latitude) [23, 24]. Taxonomic classification studies have shown multiple types of species A, C and E in the *An. minimus* complex. The habits of *An. minimus* have also been found to differ between Hainan Island and other provinces of China [23, 25, 26]. *An. minimus* was a domestic mosquito species and the main malaria transmission vector south of 25°N in China, such as in Yunnan and along the coast of Hainan. Its malaria transmission efficacy was gradually weakened north of 25° N. Therefore, *An. minimus* was the primary malaria vector in the mountainous and hilly areas of southern China [27]. *An. minimus* is a main malaria vector in the hilly and forested regions, effective control is based on usage of LLIN and IRS at small villages and unincorporated communities.

*An. dirus* is an anthrophonic species mainly distributed in Hainan and has also been found in southern Yunnan and southwest Guangxi [28, 29]. However, *An. dirus* has only been confirmed to be a crucial malaria transmission vector in Hainan’s central and south mountainous areas. Historical data from 1336 female *An. dirus* collected from human rooms at five representative sites showed an infection rate of 2.19–7.01% in 1957. Meanwhile, the highest sporozoite infection rate was recorded as 11.2% in 1962 [30]. In 1965, a survey found that the incidence of malaria in areas with *An. dirus* was 42.2–55.2% in Qiongzhong, Hainan, while the incidence rate was only 4.4–6.0% in an area without *An. dirus* [31]. This evidence indicated that the distribution of *An. dirus* was closely related to the endemic. In recent years, similar to *An. anthropophagus* and *An. minimus*, *An. dirus* has a gradually reduced distribution range. Since 2010, *An. dirus* has only been detected in Hainan Province. The unconventional biting rhythm and outdoor resting behavior of *An. dirus* challenged the effectiveness of the most commonly employed control measures. Comprehensive strategies, including environmental improvements and personal protection through the use of ITNs, LLINs or other protection products, was highly recommended.

### Malaria vector control strategies during the endemic transmission stage

According to the epidemiological characteristics of malaria throughout different historical periods, the course of malaria control in China could be classified into the following stages: the focal investigation and prevention stage (1949–1959); the severe epidemic stage (1960–1979); the incidence rate-declining stage (1980–1999); and the control/pre-elimination stage (2000–2009) [11, 32, 33].

Focal investigation and prevention stage (1949–1959): at this stage, malaria was endemic in most areas of China, but data on plasmodium, vectors, epidemiology, and regional distribution were scarce. With the establishment of the professional malaria prevention and control institutions at the national and provincial levels, as well as the baseline surveys of protozoan species, vectors, and epidemiology studies, the malaria-endemic areas and the initial vector distributions have been preliminarily determined.

Severe epidemic stage (1960–1979): The malaria prevalence rate was still high at this stage, but preliminary investigation and prevention and control allowed China to gradually perform planned and comprehensive prevention and control measures. Different prevention and control measures have been adopted in different epidemic areas. Specific technical issues and requirements such as vector control have been clarified. In the southern regions where *An. minimus* was the primary vector, comprehensive prevention and control strategies were adopted, which focused on both vector control and infection source elimination. For instance, IRS with DDT combined with case management for malaria prevention and control was promoted. In the northern region where *An. sinensis* was the primary vector, a prevention and control strategy that focused on eliminating the source of infection was adopted. In addition, all provinces have combined farmland water conservancy construction and patriotic sanitation campaigns to mobilize the masses to take adequate control on the vectors according to local conditions.

Incidence rate-declining stage (1980–1999): After comprehensive prevention and control in the early stage, the prevalence of malaria in China was significantly reduced. The malaria control strategy at this stage was based on the guidance of local governments and expert recommendations. In the endemic areas where *An. sinensis* was the dominant vector, a comprehensive control strategy based on infection source management was adopted. In the endemic regions where *An. minimus* or *An. anthropophagus* was distributed, the strategy of vector control supplemented with management of infection sources was adopted by implementing DDT indoor spraying once or twice a year (covering more than 10 million people, once a year in low endemic areas and twice a year in high endemic areas) combined with ITNs (covering more than 5 million people). In areas where *An. dirus* was distributed, a comprehensive strategy focusing on reducing breeding sites, such as environmental governance in stockade villages in forested areas, combined with infection source management was adopted. Furthermore, the technical and implementation plans for vector control, monitoring, and training were also formulated for the first time in 1983.

Control/pre-elimination stage (2000–2009): Compared with the previous epidemic stages, the control/pre-elimination stage had a significantly reduced incidence rate, although the epidemic situation rebounded in sporadic areas. In some provinces with residual malaria transmissions and epidemic resurgences, with the support of the Global Fund, environmental transformation and vector control strategies were deployed in falciparum malaria-endemic areas, such as the border areas of Yunnan and the mountainous regions in central and southern Hainan. Core vector control strategies, monitoring, and evaluation were also adopted in the areas in central China where the epidemic has rebounded. Furthermore, the implementation of IRS and LLINs has played a significant role in successfully curbing the rise of malaria in China. These strategies provided strong support for the formulation of the national malaria elimination action plan (Table 1).

**Table 1:** Malaria stages and vector control strategies in China

Stages of malaria	Approximate number of cases reported	Characteristics	Goal	Role and features of vector control strategies
Focal investigation and control (1949–1959)	32 (millions)	Endemic with scarce data	To determine prevalence, parasites and vectors and reduce the mortality and morbidity in focal areas	Baseline surveys of vectors Control trials
Severe epidemic (1960–1979)	183 (millions)	Pandemic of vivax malaria in central China	To control the pandemics	Mass IRS with DDT as a core in areas with endophilic, endophagic and anthropophilic vectors Mass IVC supplemented in exophilic, exophagic and zoophilic vectors
Incidence rate declining (1980–1999)	13 (millions)	Continuous decline of prevalence	To continue the reduction of transmission	IRS with DDT and ITNs with Pyrethroid insecticides as a core in areas with endophilic, endophagic and anthropophilic vectors IVC supplemented in exophilic, exophagic and zoophilic vectors Reducing breeding sites
Control/pre-elimination (2000–2009)	350 (thousands)	Resurgence of vivax malaria in central China	To roll back resurgence and transform control programme to elimination	IRS and ITNs/LLINs with Pyrethroid insecticides as a core in areas with endophilic, endophagic and anthropophilic vectors IVC supplemented in exophilic, exophagic and zoophilic vectors Vector surveillance Larvae control
Eliminating (2010–2020)	38 (thousands)	Sporadic transmission and imported infections	To achieve elimination	IRS in Foci and ITNs/LLINs among high risk population with high-efficacy Pyrethroid insecticides as a core in areas with endophilic, endophagic and anthropophilic vectors IVC supplemented in exophilic, exophagic and zoophilic vectors Vector distribution prediction
Post-elimination (2020-present)	< 1000 (imported cases only)	Risk of re-transmission	To prevent re-transmission caused by imported malaria	IRS in Foci and ITNs/LLINs among high risk population with high-efficacy Pyrethroid insecticides as a core in areas with endophilic, endophagic and anthropophilic vectors IVC supplemented in exophilic, exophagic and zoophilic vectors Corresponding vector control strategy based on risk stratification

### Malaria vector control strategies in the elimination stage

To actively respond to global malaria eradication initiative, 13 ministries and commissions, including the former Ministry of Health, jointly issued the *China Malaria Elimination Action Plan (2010–2020)* in 2010 and proposed that malaria would be eliminated in the country by 2020 [4, 31]. According to the reported incidence rate of indigenous malaria cases from 2006 to 2008 in China, all of the counties were divided into four tiers [11]. Although the classification was based on incidence, the malaria prevention and control strategies corresponding to each tier integrated the vector control strategies effectively. For example, for counties of tier 1, with a distribution of *An. dirus*, *An. minimus*, and *An. anthropophagus*, the provincial disease prevention and control agency was responsible for the unified procurement and provision of insecticides and LLINs. The county-level disease prevention and control agency was responsible for organizing the health workers, village doctors, and villagers’ committees to distribute LLINs and ITNs before the transmission season of each year. At the same time, many counties have combined patriotic sanitation campaigns and new rural construction to implement environmental renovation and governance, as well as encouraging residents in endemic areas to use protective measures, such as repellents, mosquito coils, mosquito nets, and screens, to reduce human and mosquito contact and strengthen personal protection.

In addition, a new “1-3-7” case- and focus-based surveillance and response approach was proposed for the elimination of malaria, especially for the “7,” in which the county-level disease control agencies were required to complete the foci investigation and disposal within 7 days [34]. “1-3-7” approach was successfully implemented with the effectiveness of > 95% in China [34, 35]. The approach further clarified the responsibilities, standardized the process, advanced the timeliness and effectiveness of vector control work related to the targeted foci, and effectively prevented the transmission of malaria.

### Malaria vector control strategies in the post-elimination stage

Following elimination, the vector control strategy is tailored and adopted into the post-elimination stage, during which the foci are still targeted and vector control measures are implemented in response to the emergencies. IRS is undertaken to remove infectious adult *Anopheles* mosquitoes in all houses within 500 m radius around a confirmed case of malaria. Once an outbreak occurred, resident houses and livestock should be treated with IRS, which could be extended to the neighboring villages if necessary. Health education for the affected population includes educating the masses to increase their awareness of self-protection, avoid going out in the wild or the field without any protection, and promote the use of mosquito nets, screens, and other anti-mosquito facilities to reduce the likelihood of mosquito bites.

In 2020, the National Health Commission, Customs, and other departments jointly devised the *Administrative Measures for Preventing Re-establishment of Transmission by Imported Malaria*, and the Chinese Center for Disease Control and Prevention (China CDC) devised the *Technical Guideline for Preventing Re-establishment of Transmission by Imported Malaria*, with the aim to prevent re-transmission, reduce and avoid malaria deaths, and consolidate the achievements in eliminating malaria. According to factors such as the distribution of malaria vectors in different regions, the *Plasmodium* species of imported cases, and the transmission conditions and pathways, the risk of re-transmission in the previous malaria-endemic areas of China was stratified by counties, including those with re-transmission risk in cross-border regions, those with risk of re-transmission of multiple parasites, those with re-transmission risk of vivax malaria, and those with potential re-transmission risk (counties with potential re-transmission risk of various parasites and counties with potential re-transmission risk of *vivax* malaria). At the same time, it was emphasized that the re-transmission risk stratification is dynamically changing. Each province should make dynamic adjustments based on annual imported cases and vector-monitoring results. The National Health Commission (NHC) should conduct a risk assessment and risk stratification adjustment every 3 years (Additional file 2: Fig. S2). At the same time, based on the risk stratification, countermeasures are devised in different stratified areas by clarifying the activities to be carried out and the entities responsible for them. In addition, it should also be emphasized that CDCs at all levels continued to pay attention to the changes in the mosquito vector density, ecological habits, and susceptibility to insecticides to further provide supporting data for the effective implementation of vector control strategies.

## Special issues

### Insecticide-impregnated mosquito nets

Since the 1970s, China has used pyrethroid insecticide-treated nets, especially those containing deltamethrin and other pyrethroid insecticides, as one of the main vector control measures in malaria control [36]. Since 2001, ITNs/LLINs and IRS have played an essential role in the control of the *falciparum* malaria epidemic in China. Since 2010, ITNs/LLINs have been used as a critical auxiliary measure for vector control in Yunnan, Hainan, and other regions to eliminate malaria. For example, in Yunnan Province, 217,919 LLINs/ITNs were used in 2011 and 140,567 were used in 2012. The number of LLINs/ITNs used for vector control gradually decreased after 2013. In Hainan Province, from 2011 to 2018, 106,845 ITN or LLIN were used in the province. Since 2010, China has issued nearly 3.33 million ITNs or LLINs (Table 2).

**Table 2:** Number of insecticide-treated mosquito nets (ITN/LLIN) distributed in 2010–2019 and the number of populations protected by indoor residual spraying

Year	No. of ITN/LLIN	No. of protected populations
2010	1,091,528	2,456,148
2011	1,840,792	1,043,963
2012	251,555	1,092,158
2013	58,874	447,639
2014	19,899	504,936
2015	29,611	1,697,188
2016	26,562	272,108
2017	11,349	352,732
2018	5987	161,224
2019	1807	206,599
Total	3,337,964	6,098,574

### Indoor residual spraying (IRS)

Since the 1980s, IRS has been one of the main vector control measures in *falciparum* malaria-endemic areas such as central China and some southern provinces [3, 37]. Following malaria elimination in China, IRS was mainly used for foci disposal to prevent transmission. The strategy was primarily used to remove infectious *Anopheles* mosquitoes at foci that have already established transmission. IRS was also adopted in areas where transmission was possible to remove potentially infectious adult *Anopheles* mosquitoes. The insecticides used in IRS were mainly pyrethroid insecticides, such as lambda-cyhalothrin suspending agents and lambda-cyhalothrin suspending agents. Since 2010, the number of people protected by IRS in China has reached more than 6.09 million (Table 2).

### Larvae control

Larvae control was a critical supplementary measure for malaria control in both the malaria control and elimination stages in China [38]. Since the 1970s, China performed a series of on-site biological anti-mosquito studies in certain areas. The unique Jingdrosuo nematode species found in Anhui province was used to infect the larvae of *An. sinensis*; it has been used for small-scale larval control in Hubei and Guangdong. Other species, such as *Bacillus thuringiensis israelensis* (Bti) and *Bacillus sphaericus* (Bs), were also widely used for larvae control [39]. For example, Henan Province performed a small-scale field study involving the application of Bs in the malaria control stage, with great success.

Additionally, Guiyang conducted a series of studies on the mosquito-killing effect of Pythium and other entomogenous fungi. In addition, other experiments and field applications involving killing larvae using loach, mosquito-eating fish (wicker fish), rice field eels, wild crucian carp, and ziling goby were conducted in many other places in China. The use of intermittent irrigation in rice fields for mosquito larvae control has also achieved some success in places such as Hainan, Jiangsu Province.

### Vector surveillance

In the malaria elimination stage, vector surveillance provided the scientific basis to correctly judge the type of foci and implement corresponding vector control strategies [40]. China has established a relatively comprehensive vector surveillance network. In the pre-elimination and elimination stages, national malaria vector surveillance sites were set up in historical malaria-endemic provinces and some provinces at risk (Additional file 3: Fig. S3). At the same time, all provinces were encouraged to establish provincial vector surveillance sites based on actual incidences. A survey of the vector population and density was recommended to be performed annually. Batches of sites were selected for monitoring insecticide resistance. In addition, customized malaria vector investigations were performed in areas reported to have imported malaria cases. For example, in where imported malaria cases occurred or a smart of returnees back from high-risk countries, vector investigation should be performed if without entomological data in the past 3 years. In addition, the customs system has also been included in the Chinese vector surveillance network. For example, customs at various levels performed mosquito monitoring at border ports and inbound vehicles and performed targeted prevention and control measures.

Currently, data from surveillance sites in China have shown that the main vector species were *An. sinensis*, *An. anthropophagus, An. minimus,* and *An. dirus. An. sinensis* had the most widespread distribution. Indeed, since 2010, *An. sinensis* has been found in most surveillance sites. By contrast, *An. anthropophagus* has only been observed in Guizhou, Hainan, and Liaoning provinces. *An. minimus* has only been found in Yunnan and Hainan provinces, and *An. dirus* has only been found in Hainan Province. *Anopheles maculatus*, a malaria vector found in India, Malaysia, Indonesia, and the Philippines, has also been found in Tibet. From 2010 to 2019, 11 malaria vector species were intercepted or monitored at Chinese ports. Among them, *Anopheles jeppel,* intercepted from ships entering the country, was not only a malaria vector in some parts of Southeast Asia but also a potential malaria vector in the mountainous areas of southern China.

### Malaria vector investigation on foci

In the elimination stage, with the yearly decreased number of indigenous malaria cases, no large-scale vector control strategy was implemented. The focus was mainly on the vector control in the targeted areas. The county-level (CDC) where the vector surveillance sites were located performed annual vector population and density surveys. For counties without vector data over a 3-year period, the light trap method was used to conduct vector population surveys. The human-baited method was used to conduct density surveys to provide a scientific basis to determine the type of foci and establish effective foci disposal measures. Once the foci occurred and were identified, especially the occurrence of suspected indigenous infection, secondary transmission, or other unexplained cases under certain circumstances, a large-scale survey of vector-breeding sites and vector density was conducted to provide a scientific basis for focus investigations in order to better manage the epidemic and reduce transmission risk.

### Insecticide resistance

The four insecticides used frequently in China included organochlorine, organophosphorus, carbamate, and pyrethroids. According to the bioassay method for detecting mosquito resistance (GB/T26347-2010), insecticide surveillance sites were set in areas where *An. dirus*, *An. minimus*, and *An. anthropophagus* were distributed, or where *An. sinensis* was distributed at a high density every two years to determine the resistance status. The results of the resistance surveys in Sichuan, Yunnan, Hainan, and Shandong provinces showed that *An. sinensis* was only sensitive to 5.0% malathion and 0.05% deltamethrin in Hainan Province. In contrast, *An. sinensis* in other provinces reached a level of resistance to all types of tested insecticides defined by WHO. Similarly, surveillance results in Henan and Hainan provinces in 2017 showed that, except for 5.0% malathion, *An. sinensis* reached the same resistance level to deltamethrin as to the other three insecticides determined by WHO. In 2018, the insecticide survey launched in Hainan, Henan, Guizhou, Jiangsu, Hubei, Guangdong, Liaoning, and Shandong showed that, except for *An. sinensis* in Guangdong Province that was suspected to be deltamethrin-resistant, *An. sinensis* in other provinces was found to be deltamethrin-resistant.

### Scientific research on malaria vector

Given that accurate species identification is crucial for all studies and surveillance activities on field populations of vectors, researchers have since conducted studies on the *Anopheles* complex [27]. For example, the *An. dirus* complex is composed of eight sibling species. *An. dirus* A in Hainan and *An. dirus* D in Yunnan Province were determined by molecular experiments [41, 42]. In addition, through the domestication and conservation of *Anopheles* collected from the wild, researchers have observed the laboratory breeding of some major vectors except for *An. minimus*, and established laboratory infection models to study the effects of *Anopheles* on malaria in different regions. The susceptibility of plasmodium strains and different vector species could be used to clarify the vector capacity of different vectors [30, 43]. LH Tang published an academic monograph to guide the control of *An. anthropophagus* in China, which included life history, morphology, ecology, distribution, role in disease transmission, control and monitoring of the species. It played a direct guiding role in the control and prevention of malaria and filariasis in China and has important reference value for controlling other vector-borne diseases [20]. The release of *An. sinensis* and other species’ genomes, as well as the functional research of related immune- and resistance-related genes, have further refined vector control.

### Supervision and assessment

Supervision of vector control has been performed at all CDC levels in various regions in China. For example, in the early stage of malaria elimination, the health administrative departments in endemic areas such as Yunnan and Hainan conducted on-site supervisions of the distribution and use of LLIN from the CDC. In addition, many provinces organized the disease control agencies to facilitate the correct disposal of IRS. The higher authorities conducted on-site investigations and supervision and reported indicators such as the number of ITN/LLIN used and the number of IRS-protected populations in the annual malaria report. To improve the quality of *Anopheles* vector monitoring, the National Institute of Parasitic Diseases (NIPD) and China CDC, in accordance with the requirements of the *National Work Plan for Anopheles Vectors Monitoring Sites in the Phase of Malaria Elimination*, organized and performed surveys on *Anopheles* vector monitoring and visited vector monitoring sites in various provinces. The NIPD performed research and supervision by report reading, data review, personnel interviews, sample testing, and spot checks, and regularly reported the results to regulate the rectification process. In addition, some other inspection and quarantine departments, such as the General Administration of Customs and directly affiliated customs, regularly organized supervision and guidance on the vector monitoring in ports or customs inspection areas.

### Training

Under the organization and coordination of the National Health Commission, all levels of China’s CDCs have professional personnel engaged in appropriate training and technical supports to ensure the quality of vector monitoring and control. The NIPD organized professional and technical personnel to conduct training on species identification and investigation techniques, while the CDC of each province organized technical training for professional and technical personnel. Since 2010, most provinces have held vector monitoring technology training courses annually, with more than 2,000 people attending each year. The customs system also actively participated in the CDC’s training course or conducted its own training, including morphological identification of *Anopheles*.

### Support mechanism

The main sources of financial investment for malaria elimination came from government expenditures. Vector control was a component of this scheduled investment. After malaria elimination, the central government continued to maintain funding for sustaining the malaria-free status. In 2021, the Ministry of Finance and the National Health Commission will dedicate 67.65 million Yuan to malaria prevention and control funds in the preliminary public health services for operation and management, of which 5 million will be used for vector control. The central essential public health services will continue to maintain malaria prevention and control funding from 2022 to 2023. Consumables and equipment related to vector control will meet the needs of vector control during post-elimination phase. Pesticides and equipment for IRS are currently equipped in county and township health centers or community health service centers. Professional and technical personnel implemented health education for the affected population before spraying, during which they introduced the importance and significance of spraying. During the spraying process, personnel wore protective masks and clothing to ensure personal protection.

### Experience and lessons learned

In response to the different malaria-endemic situations and vector distributions, China adopted different prevention and control measures. Around 1980, case managements and vector control were mainly adopted for endemic areas targeting *An. sinensis*. In endemic areas where *An. anthropophagus* and *An. minimus* were sympatrically distributed, the distribution of ITNs and IRS to kill mosquitoes was mainly adopted, followed by case management. In areas where *An. dirus* was the dominant species, environmental modification was performed to eliminate mosquito breeding sites and prevent transmission as supplementary strategy. Following the long-term and unremitting adoption of comprehensive anti-malarial and vector control strategies of the 1990s, China’s economy has developed rapidly. Living standards, environments, and sanitary conditions have improved. Human-mosquito contact and the frequency of malaria infection have been reduced. These improvements were particularly significant in areas where *An. sinensis* was the malaria vector. At the same time, large-scale farmland water conservancy construction, changes in farming systems, the use of chemical pesticides in rice fields, and development and production around villages have also contributed to a reduction in the breeding environment of the *Anopheles* mosquito. As a result, the distribution range and number of infections have been reduced and the malaria transmission was decreased, which led to a fundamental change in the conditions of malaria transmission in some areas. China’s experience in vector control from the malaria epidemic to its elimination are summarized as follows: (1) baseline surveys were initiated to establish the baseline data of the vector and to clarify the prevention priorities and targets; (2) targeted and adjusted vector control strategies were conducted in different regions according to the local epidemic characteristics and different vectors; (3) scientific and efficient vector control strategies were facilitated through promoting vector research; (4) overall economic and social development have promoted environmental improvement, personal protection, and health care; and (5) prediction of the vector distribution based on risk assessment strategies, allowing maintenance of malaria free in risk areas.

### Challenges ahead

Although China has achieved elimination of malaria, it still faces many challenges in malaria vector control as follows:

### Border areas with complex malaria vectors and high epidemic potential

Border areas, especially Yunnan, neighboring Myanmar, Laos, and Vietnam, with a borderline of 4060 km, an altitude of 6740 m, and a minimum of 76.4 m, have complex geographic environments. Multiple climate types coexist in these areas. The natural conditions are suitable for the prevalence of vector-borne diseases, such as malaria and dengue fever [44]. The border areas have various malaria transmission vectors, especially *An. dirus* and *An. minimu*s, with high vector competence that was aggravated by the mixed epidemic of *falciparum* and *vivax malaria* [45]. This represents a key challenge to malaria prevention and control in China, even in the post-elimination stage. In addition, as mosquitoes know no borders, health workers should be alerted to constantly monitor and detect potential sporozoite positive *Anopheles* mosquitoes in the border areas to prevent secondary transmission from these imported mosquitoes.

### Insecticide resistance

Insecticide resistance is a decisive factor in the core vector control strategy [[46, 47]. In the absence of effective vaccines and timely supply of antimalarial drugs, the control of the spread of malaria still largely depends on vector control. At present, *An. sinensis*, the most widely distributed *Anopheles* in China, has shown resistance to various insecticides [48]. In addition, due to the widespread and excessive use of agricultural pesticides, other *Anopheles* such as *An. minimus* are also developing resistance to one or more insecticides, which will seriously threaten the effectiveness of vector control such as IRS and LLNs. Both researchers and policymakers must consider an updated and effective plan to face this challenge, even during the post-elimination stage.

### Transformation of ecological habits of the vector and their susceptibility to parasite

In the past 20 years, with rapid economic development and changes in cultural and social environments, the distribution of vector and ecological habits has also undergone significant changes [[49]. The most obvious change was the considerable reduction in the distribution of *An. anthropophagus* and *An. dirus*. *An. anthropophagus* was once the dominant mosquito species in Jiangsu Province and some other areas, however it was difficult to find its traces again in recent years. In addition, in the malaria elimination stage, foci investigations and disposals are needed to be carried out when malaria cases reported, which include vector control at every focus that had the possibility of re-transmission. However, current knowledge of the possibility of transmission is based on the previous susceptibility of local vectors to malaria parasites. There are no relevant data on the susceptibility of local vectors to imported malaria parasites. Since 2017, China has had no reports of indigenous malaria cases, but there were still thousands of imported cases each year. The imported cases mainly occurred in foreign workers traveling to and from malaria-endemic areas such as Africa and Southeast Asia. As malaria vectors still exist in the previous malaria-endemic regions, a resurgence of the source of infection in these areas is likely to trigger local malaria transmission.

### Maintenance of malaria detection capacity in health workers

After the elimination of malaria, the malaria vector continued to exist. As long as there is imported malaria, the risk of new transmission still exists [8, 50]. At the same time, changes in environmental climate and human and animal activities will affect the distribution of *Anopheles* and its blood-seeking habits. Therefore, long-term vector-monitoring capabilities are still needed to provide a sufficient and reliable basis for devising and implementing vector control strategies. However, with the decline in malaria prevalence in recent years, vector monitoring and identification capabilities have been significantly weakened.

### Translation and scale-up of novel vector control strategies

Integrated Vector Management (IVM) is a rational decision-making process that is used to optimize the use of resources for vector control [51]. Therefore, WHO encourages the use of IVM to improve the cost efficiency of vector control measures. At the same time, in addition to the core vector control strategies, scientists continue to develop new vector control strategies in response to insecticide resistance and other issues. With the rapid development of modern biotechnology, several safe, powerful, and efficient vector control measures have been created. For example, by using embryo microinjection to infect mosquitoes with specific Wolbachia, the number of mosquitoes could be controlled through population suppression and replacement, and the transmission of mosquito-borne diseases such as dengue fever and malaria were blocked [52]. Moreover, genetically modified (GM) mosquito deployment could suppress populations of their natural brethren. This strategy was also attempted against malaria [53, 54]. However, GM mosquitoes have triggered heated discussions regarding their unpredictable nature. Recently, an independent research team reported that some of the offspring of GM mosquitoes survived and passed their genes to the local mosquito population [55]. Furthermore, transmission-blocking malaria vaccines [56] and vector-targeted vaccines [57] might be viable options for novel malaria vector control.

## Conclusions

Vector control is a vital component of malaria prevention, control, and elimination strategies. The goal of malaria vector control is to treat all people at risk with ITNs/LLINs and IRS. This approach can prevent bites from infected mosquitoes, reduce the intensity of endemic malaria transmission in the community, and decrease the incidence and prevalence of diseases. In the process from the malaria epidemic to elimination in China, vector control played a role in both disease prevention and control. During the epidemic, the implementation of vector control strategies played a key role in protecting and preventing people at risk from the perspective of population protection. The adopted vector control strategies reduced the incidence rate as a whole and controlled the large-scale outbreak of the disease. In the elimination and post-elimination phases, the vector control strategy focused more on its preventive part to minimize the occurrence of re-transmission in the case of imported or introduced cases. From 30 million malaria cases to zero, different historical periods of malaria epidemics have witnessed the continuous evolution and improvement of vector control strategies. Vector control has played an inestimable role in achieving malaria elimination. In recent years, the sharp decline in the number of ITNs/LLINs and IRS for vector control has also reflected China’s continuous achievements in eliminating malaria. Therefore, the evolution of vector control in history has mirrored and witnessed the process of malaria elimination and will continue to support the development of a framework for malaria prevention and treatment.

## Supplementary Information

**Additional file 1: Figure S1.** Distribution map of the four major malaria vector species (*Anopheles sinensis*, *Anopheles anthropophagus*, *Anopheles minimus*, and *Anopheles dirus*) in China.

**Additional file 2: Figure S2.** Risk stratification for the re-transmission of malaria in China, 2020.

**Additional file 3: Figure S3.** Vector surveillance sites during A) the pre-elimination stage, and B) the malaria elimination stage.

## Data Availability

Not applicable.

## References

[1] Guglielmi G. Malaria cases are falling worldwide. Nature. 2019. 10.1038/d41586-019-03746-3 33268876

[2] Organization WH. World malaria report 2020: 20 years of global progress and challenges. World malaria report 2020: 20 years of global progress and challenges; 2020.

[3] Yin JH, Yang MN, Zhou SS, Wang Y, Feng J, Xia ZG (2013). Changing malaria transmission and implications in China towards National Malaria Elimination Programme between 2010 and 2012. PLoS ONE.

[4] Feng X, Levens J, Zhou XN (2020). Protecting the gains of malaria elimination in China. Infect Dis Poverty.

[5] Lobo NF, Achee NL, Greico J, Collins FH. Modern vector control. Cold Spring Harbor perspectives in medicine. 2017. 10.1101/cshperspect.a025643 PMC5749144 28507198

[6] Kalinga AK, Mwanziva C, Chiduo S, Mswanya C, Ishengoma DI, Francis F (2018). Comparison of visual and automated Deki Reader interpretation of malaria rapid diagnostic tests in rural Tanzanian military health facilities. Malar J.

[7] Chala B, Erko B, Animut A, Degarege A, Petros B (2016). Assessment of Clarias gariepinus as a biological control agent against mosquito larvae. BMC Ecol.

[8] Hii J, Hustedt J, Bangs MJ (2021). Residual Malaria transmission in select countries of Asia-pacific region: old wine in a new barrel. J Infect Dis.

[9] Choi L, Pryce J, Richardson M, Lutje V, Walshe D, Garner P. Guidelines for malaria vector control. World Health Organization. 2019:1–171.

[10] Bai L, Morton LC, Liu Q (2013). Climate change and mosquito-borne diseases in China: a review. Glob Health.

[11] Zhou X-N, Xia Z-G, Wang R-B, Qian Y-J, Zhou S-S, Utzinger J (2014). Feasibility and roadmap analysis for malaria elimination in China. Adv Parasitol.

[12] Feng X, Zhang S, Huang F, Zhang L, Feng J, Xia Z (2017). Biology, bionomics and molecular biology of Anopheles sinensis Wiedemann 1828 (Diptera: Culicidae), main malaria vector in China. Front Microbiol.

[13] Qu F, Xu S, Xu J, Xu X (2010). On the biosystematics of anopheles dirus complex (diptera: culicidae) in china. Insect Science.

[14] Feng X, Huang L, Lin L, Yang M, Ma Y (2017). Genetic diversity and population structure of the primary malaria vector Anopheles sinensis (Diptera: Culicidae) in China inferred by cox1 gene. Parasit Vectors.

[15] Pan JY, Zhou SS, Zheng X, Huang F, Wang DQ, Shen YZ (2012). Vector capacity of Anopheles sinensis in malaria outbreak areas of central China. Parasit Vectors.

[16] Li H, Yang B, Wang W, Hu H, Wang W, Wang X (1998). Observation on the infectivity of different densities of Plasmodium vivax to Anopheles sinensis. Zhongguo Ji Sheng Chong Xue Yu Ji Sheng Chong Bing Za Zhi.

[17] Gu ZC, Shang LY, Chen JS, Zheng X, Su YJ, Li AM (2001). The role of Anopheles anthropophagus in malaria transmission in in Xinyang City of Henan Province. Zhongguo Ji Sheng Chong Xue Yu Ji Sheng Chong Bing Za Zhi.

[18] Zheng X, Tang LH, Gu ZC, Zhu TH, Shi WQ, Jiang WK (2007). Morphology and habits of An. anthropophagus and its role in malaria transmission in Hengqin Island of Zhuhai City. Zhongguo Ji Sheng Chong Xue Yu Ji Sheng Chong Bing Za Zhi.

[19] Jingyuan H, Zhang. G, Liu. H. Study on the Ecology Character of Distribution and Role of Malarial Transmission in Anopheles anthropophagus in Hubei, China. Chin J Vector Biol Control. 2000:03.

[20] Tang LH. Biology and control of Anopheles anthropophagus in China [M]. Shanghai Science and Technology Press, 2008. (in Chinese)

[21] Liu C (1990). Comparative studies on the role of Anopheles anthropophagus and Anopheles sinensis in malaria transmission in China. Zhonghua Liu Xing Bing Xue Za Zhi.

[22] Liu W, Huang H, Xing C, Li C, Tan F, Liang S (2014). Identification and characterization of the expression profile of microRNAs in Anopheles anthropophagus. Parasit Vectors.

[23] Shi WQ, Zhou XJ, Zhang Y, Zhou XN, Hu L, Wang XZ (2011). An investigation on malaria vectors in western part of China-Myanmar border. Zhongguo Ji Sheng Chong Xue Yu Ji Sheng Chong Bing Za Zhi.

[24] Chen B, Harbach RE, Butlin RK (2002). Molecular and morphological studies on the Anopheles minimus group of mosquitoes in southern China: taxonomic review, distribution and malaria vector status. Med Vet Entomol.

[25] Yu G, Yan G, Zhang N, Zhong D, Wang Y, He Z (2013). The Anopheles community and the role of Anopheles minimus on malaria transmission on the China-Myanmar border. Parasit Vectors.

[26] Meide L, Xuezhong W, Tongyan Z, Du Z, Yande D, Baolin L (2008). Analysis of the relationship between density and dominance of Anopheles minimus (Diptera: Culicidae) with environmental parameters in southern Yunnan Province, Peoples Republic of China. J Med Entomol.

[27] Sawabe K, Takagi M, Tsuda Y, Tang LH, Xu JJ, Qiu CP (1996). Genetic differentiation among three populations of Anopheles minimus of Guangxi and Yunnan Provinces in the People’s Republic of China. Southeast Asian J Trop Med Public Health.

[28] Zhou HN, Lu YR (1998). Studies on geographical distribution, ecology and habits, role in Malaria Transmission of Anopheles dirus in Yunnan. Chin J Vector Biol Control..

[29] Walton C, Handley JM, Tunlin W, Collins FH, Harbach RE, Baimai V, et al. Population structure and population history of Anopheles dirus mosquitoes in Southeast Asia. 2000. 10.1093/oxfordjournals.molbev.a026377 10833203

[30] He DX, Xu ZG, Ye YY, Long ZP (1983). Experimental study on susceptibility of Anopheles minimus and Anopheles dirus to Plasmodium vivax and Plasmodium falciparum. JOGN Nurs.

[31] Feng X, Xia ZG, Feng J, Zhang L, Yan H, Tang L (2020). The contributions and achievements on malaria control and forthcoming elimination in China over the past 70 years by NIPD-CTDR. Adv Parasitol.

[32] Xia ZG, Feng J, Zhou SS (2013). Malaria situation in the People’s Republic of China in 2012. Zhongguo Ji Sheng Chong Xue Yu Ji Sheng Chong Bing Za Zhi.

[33] Zhang L, Zhou SS, Feng J, Fang W, Xia ZG (2015). Malaria situation in the People’s Republic of China in 2014. Zhongguo Ji Sheng Chong Xue Yu Ji Sheng Chong Bing Za Zhi.

[34] Cao J, Sturrock HJ, Cotter C, Zhou S, Zhou H, Liu Y (2014). Communicating and monitoring surveillance and response activities for malaria elimination: China’s “1–3–7” strategy. PLoS Med.

[35] Zhang L, Feng J, Xia ZG, Zhou SS (2020). Epidemiological characteristics of malaria and progress on its elimination in China in 2019. Chin J Parasitol Parasitic Dis.

[36] Lai S, Li Z, Wardrop NA, Sun J, Head MG, Huang Z (2017). Malaria in China, 2011–2015: an observational study. Bull World Health Organ.

[37] Scho CJ, Peijun Shi. Malaria vector control strategy. Foreign Med Sci: Parasitic Dis, 1994; 021(002):65–66.

[38] Yu S, Ji C, Zhu X, Xue J, Wang L, Wang Y (2017). Impact of Bacillus sphaericus exposure on Anopheles dirus’s fecundity and resistance development. Parasitol Res.

[39] Zhuang L, Zhou S, Wang Y, Chang M (2011). Mosquito biolarvicide production by sequential fermentation with dual strains of Bacillus thuringiensis subsp. israelensis and Bacillus sphaericus using sewage sludge. Bioresource Technol.

[40] Feng XY, Xia ZG, Vong S, Yang WZ, Zhou SS (2014). Surveillance and response to drive the national malaria elimination program. Adv Parasitol.

[41] Qi G, Cooper R, Huayun Z. Genetic identification of Anopheles anthropophagus and Anopheles Sinensis by PCR-RFLP. Chin J Zoonoses. 2002:06.

[42] Fengyi Q, Yajun M, Jiannong X, Zheming Z. Sequence differences of rDNA-ITS2 and species-diagnostic PCR assay of Anopheles sinensis and Anopheles anthropophagus from China [J]. 1998;2.

[43] Zhu G, Xia H, Zhou H, Li J, Lu F, Liu Y (2013). Susceptibility of Anopheles sinensis to Plasmodium vivax in malarial outbreak areas of central China. Parasit Vectors.

[44] Zhang SS, Zhou SS, Zhou ZB, Chen TM, Wang XZ, Shi WQ (2018). Monitoring of malaria vectors at the China-Myanmar border while approaching malaria elimination. Parasit Vectors.

[45] Chen T, Zhang S, Zhou SS, Wang X, Luo C, Zeng X (2017). Receptivity to malaria in the China-Myanmar border in Yingjiang County, Yunnan Province, China. Malar J.

[46] Brogdon WG, McAllister JC (2004). Insecticide resistance and vector control. J Agromedicine.

[47] Alout H, Labbé P, Chandre F, Cohuet A (2017). Malaria vector control still matters despite insecticide resistance. Trends Parasitol.

[48] Chang X, Zhong D, Fang Q, Hartsel J, Zhou G, Shi L (2014). Multiple resistances and complex mechanisms of Anopheles sinensis mosquito: a major obstacle to mosquito-borne diseases control and elimination in China. PLoS Negl Trop Dis.

[49] Ren Z, Wang D, Ma A, Hwang J, Bennett A, Sturrock HJ, et al. Predicting malaria vector distribution under climate change scenarios in China: challenges for malaria elimination. Sci Rep. 2016;6. 10.1038/srep20604 PMC4751525 26868185

[50] Sriwichai P, Karl S, Samung Y, Kiattibutr K, Sirichaisinthop J, Mueller I (2017). Imported Plasmodium falciparum and locally transmitted Plasmodium vivax: cross-border malaria transmission scenario in northwestern Thailand. Malar J.

[51] Golding N, Wilson AL, Moyes CL, Cano J, Pigott DM, Velayudhan R (2015). Integrating vector control across diseases. BMC Med.

[52] Ross PA, Turelli M, Hoffmann AA (2019). Evolutionary ecology of wolbachia releases for disease control. Annu Rev Genet.

[53] Marshall JM, Touré MB, Traore MM, Famenini S, Taylor CE (2010). Perspectives of people in Mali toward genetically-modified mosquitoes for malaria control. Malar J.

[54] Cisnetto V, Barlow J (2020). The development of complex and controversial innovations. Genetically modified mosquitoes for malaria eradication. Res Policy.

[55] Servick K (2019). GM mosquito study draws fire. Science.

[56] Challenger JD, Olivera Mesa D, Da DF, Yerbanga RS, Lefèvre T, Cohuet A (2021). Predicting the public health impact of a malaria transmission-blocking vaccine. Nat Commun.

[57] Manning JE, Oliveira F, Coutinho-Abreu IV, Herbert S, Meneses C, Kamhawi S (2020). Safety and immunogenicity of a mosquito saliva peptide-based vaccine: a randomised, placebo-controlled, double-blind, phase 1 trial. Lancet (London, England).

