# Supplementary figures and images for: Vector control in China, from malaria endemic to elimination and challenges ahead
Source: Infect Dis Poverty. 2022 May 13;11:54. doi: 10.1186/s40249-022-00971-3 (PMC9102289; doi:10.1186/s40249-022-00971-3)

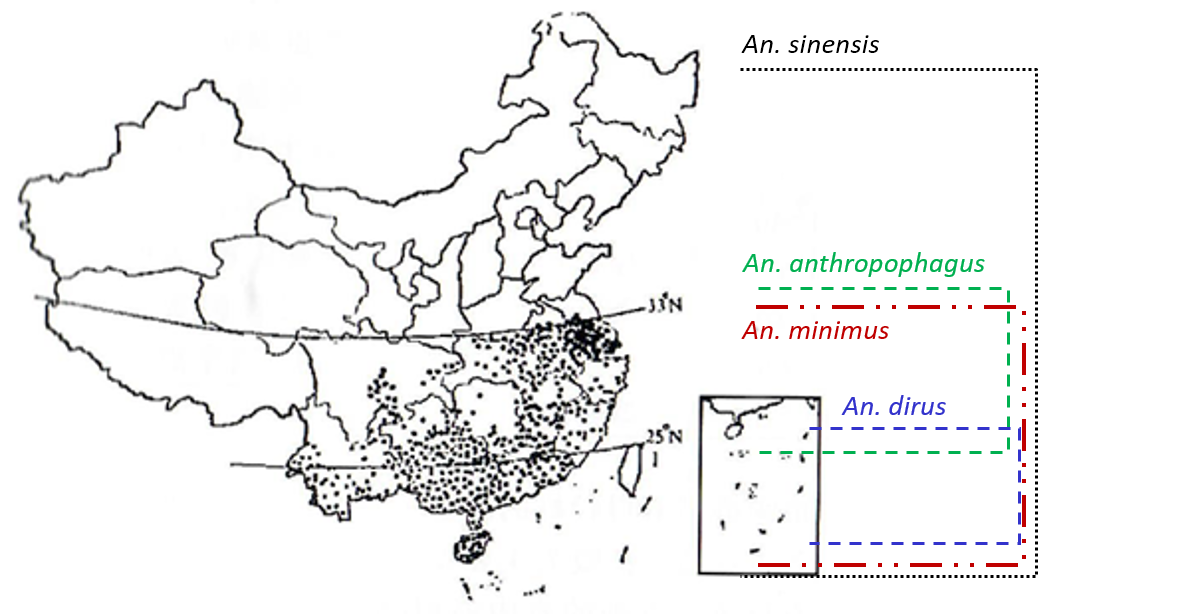

Supplement: Supplementary file 1 — Additional file 1: Figure S1. Distribution map of the four major malaria vector species (Anopheles sinensis, Anopheles anthropophagus, Anopheles minimus, and Anopheles dirus) in China. [file 40249_2022_971_MOESM1_ESM.tif]

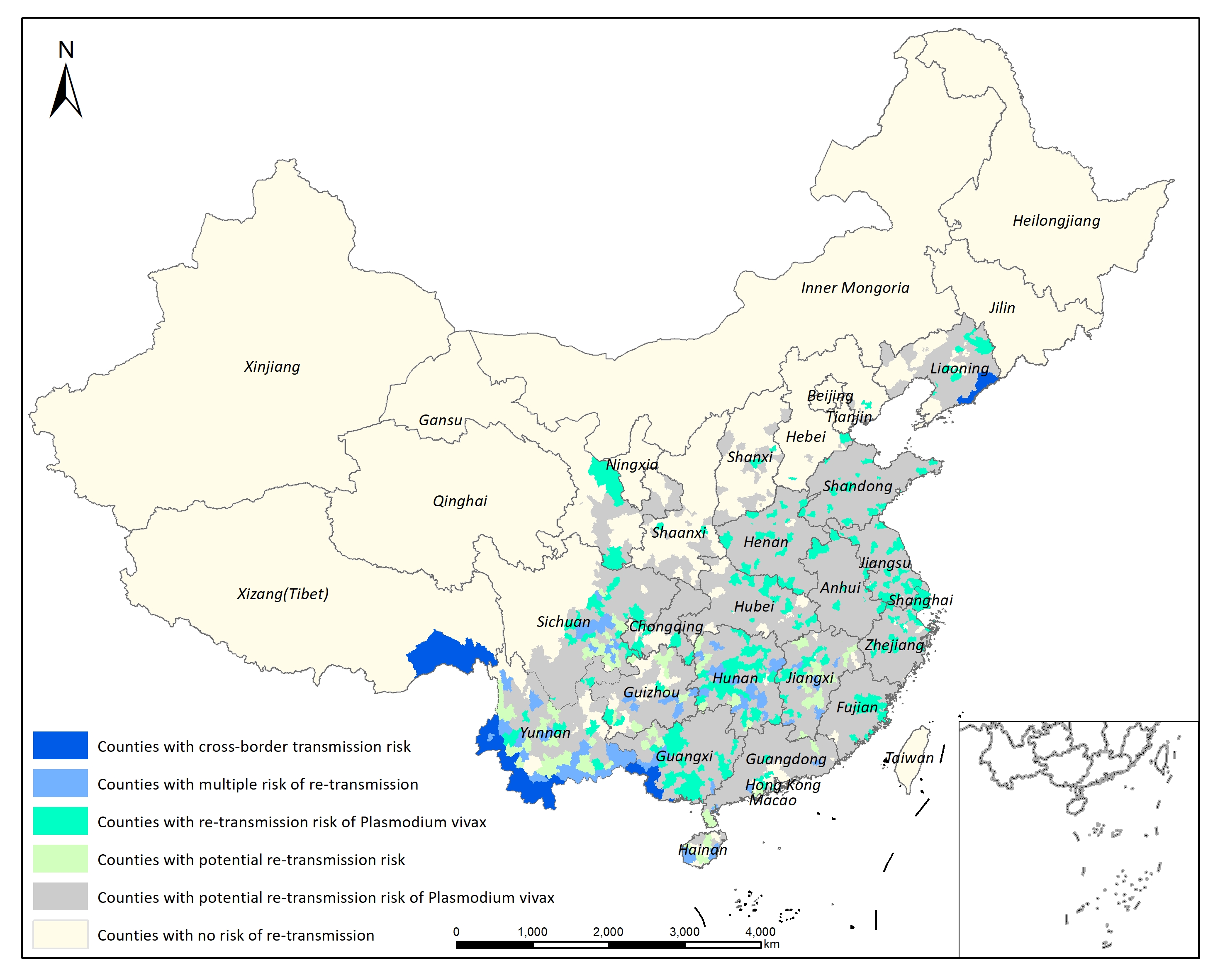

Supplement: Supplementary file 2 — Additional file 2: Figure S2. Risk stratification for the re-transmission of malaria in China, 2020. [file 40249_2022_971_MOESM2_ESM.jpg]

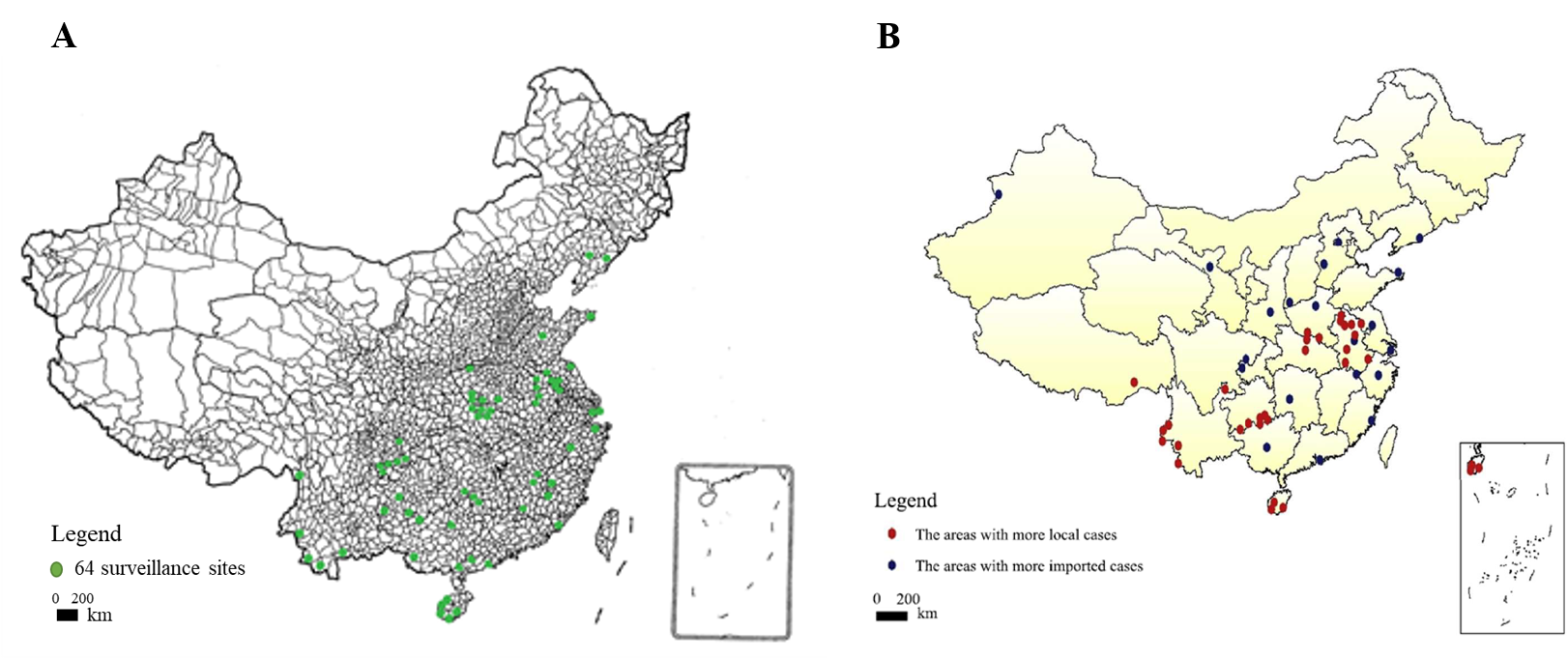

Supplement: Supplementary file 3 — Additional file 3: Figure S3. Vector surveillance sites during A) the pre-elimination stage, and B) the malaria elimination stage. [file 40249_2022_971_MOESM3_ESM.tif]
